# Internet use and electronic gaming by children and adolescents with emotional and behavioural problems in Australia – results from the second Child and Adolescent Survey of Mental Health and Wellbeing

**DOI:** 10.1186/s12889-016-3058-1

**Published:** 2016-05-13

**Authors:** Wavne Rikkers, David Lawrence, Jennifer Hafekost, Stephen R. Zubrick

**Affiliations:** Telethon Kids Institute, The University of Western Australia, PO Box 855, West Perth, WA 6872 Australia; Faculty of Education, The University of Western Australia, Perth, Australia

**Keywords:** Mental disorders, Internet use, Electronic gaming, Internet problem behaviours, Children and adolescents

## Abstract

**Background:**

Concerns have been raised of a potential connection between excessive online activity outside the academic realm and increased levels of psychological distress in young people. Young Minds Matter: the second Australian Child and Adolescent Survey of Mental Health and Wellbeing provides estimates of the prevalence of online activity and allows an exploration of associations between this activity, a range of mental disorders, socio-demographic characteristics and risk taking behaviour.

**Methods:**

Based on a randomized nationally representative sample, a household survey of mental health and wellbeing (Young Minds Matter) was conducted in 2013-14. Interviews were conducted with 6,310 parents and carers of 4–17 year-olds (55 % response rate), together with self-report questionnaires completed by 2,967 11–17 year-olds in these households (89 % response rate). The survey identified a range of mental disorders and emotional problems using a variety of diagnostic tools, with the self-report including questions about use of the Internet and electronic games. Five behaviours were measured related to this activity, with ‘problem behaviour’ being defined as exhibiting at least four out of five behaviours.

**Results:**

Levels of Internet use (98.9 %, CI 98.5–99.3 %) and electronic gaming (85.3 %, CI 83.9–86.6 %) were high, and 3.9 % (CI 3.2–4.6 %) of young people reported problem behaviour. The proportion of girls with very high levels of psychological distress and problem behaviour (41.8 %,CI 28.8–54.9 %) was twice that for boys (19.4 %, CI 7.7–31.1 %). Those engaging with a range of risk factors reported higher prevalence of problem behaviour than others. Youth who suffered from emotional problems or high levels of psychological distress spent the most time online or playing games. Multivariate analysis showed associations with problem behaviour and having attempted suicide, experiencing high to very high levels of psychological distress, using alcohol, and living in a poorly functioning family. It was not possible to determine the direction of the associations.

**Conclusion:**

There are links between problem behaviours associated with Internet use and electronic gaming, and mental disorders and risk-taking behaviour in young people. Further studies are required to determine whether these are precursors or sequelae.

**Electronic supplementary material:**

The online version of this article (doi:10.1186/s12889-016-3058-1) contains supplementary material, which is available to authorized users.

## Background

Children growing up in Australia today have never known a world without the Internet and electronic games and, for them, the term ‘being connected’ has an entirely different connotation than it did for their parents as children. Moreover, a new pedagogical model has evolved in Australia which incorporates regular computer use into the school curriculum from pre-primary onwards, with 92 % of 5–14 year olds spending time in online educational activities [1]. This adoption of digital-based learning activities effectively gives children an imprimatur to turn to the Internet as a firsthand source of knowledge and as a ‘legitimate’ way to spend their time. Nevertheless, concerns have been raised of a potential connection between problematic online activity outside the academic realm and increased levels of psychological distress in young people [2–6]. Young Minds Matter: the second Australian Child and Adolescent Survey of Mental Health and Wellbeing 2013–14 (i.e. Young Minds Matter - YMM) captured information from a large, nationally representative sample of young people and their parents or carers which included, inter alia, information about their mental disorders, Internet use, electronic gaming, and risk taking behaviour [7]. We present here results from the survey which are related to Internet use and electronic gaming behaviour and its correlates.

According to the Children’s Rights Report 2014, children see digital citizenship as ‘fundamental to their well-being’ [8]. The boundary between online and off-line is often blurred for them [9] and they tend to see digital access as a natural and integral part of their day to day existence and their fundamental right. [10] In 2013 some 81 % (14.7 million) of Australians had a home Internet connection [11] and just over 2 million Australian households with children under the age of 15 accessed the Internet on a daily basis [12]. In addition, 98 % of households with children younger than 18 years had access to computer games, as growing numbers of parents see electronic gaming as a way to spend time with their children, educate them in how to solve problems through strategizing, and as a means of behaviour reinforcement [13]. However, data drawn from the AU Kids Online study [14] shows that Australian children were more likely than any of the children in the 25 European countries surveyed in the EU Kids Online survey [15] to have experienced one or more problem behaviours relating to Internet use (50 %-Australia; 29 %-Europe). This indicates a potential problem of significance.

Almost since the advent of the Internet, researchers have explored the roles that both the Internet and electronic gaming play in the mental health of children and adults alike [16–20]. It has been found that there are young people who find going online or electronic gaming to be addictive, to the extent that it becomes harmful to their mental health and causes negative impacts on their life, such as taking time away from other activities like school work or interaction with family and friends, which in turn, can lead to feelings of distress and loneliness [6, 15]. In recognition of gaming’s deleterious effects, Internet Gaming Disorder has been included in the latest edition of the *Diagnostic and Statistical Manual of Mental Disorders* (DSM-5) as a condition meriting further research [21]. Others have proposed that Internet addiction should also have been considered for inclusion in DSM-5 as it exhibits similar properties to a compulsive-impulsive disorder [22]. Although there seems little dispute that problems exist with respect to excessive use of, or addiction to the Internet and/or electronic gaming [17, 20, 22, 23], definitional confusion and inconsistencies across diagnostic criteria have plagued efforts to formalize any disorder and it remains a controversial issue and one for which there is no ‘gold standard’ according to Kuss et al. [24] Terms are many and varied – with respect to YMM results, we use the term problematic or problem Internet use and electronic gaming behaviour and we define this later in the paper.

There are numerous studies establishing comorbidity between the condition characterized as Internet Addiction (IA) in adolescents and mental disorders including, but not restricted to, depression, Attention Deficit and Hyperactivity Disorder (ADHD), and suicidal behaviour [3, 18, 25–29]. Indeed, research in the United States has found that 86 % of people with IA suffered comorbidity with another DSM-IV disorder [22]. While it is still unclear as to the causal nature of these relationships, that is, whether having a mental health disorder leads to over-use of the Internet or vice versa [4, 14, 17, 28, 30–34], the significance of the links is sufficient to warrant concern. The question as to whether the Internet is soothing for, or conversely contributes to, psychological distress is vexing and has been described elsewhere as the Internet Paradox [35]. In a follow-up study on this phenomenon the researchers found that introverts who were greater users of the Internet showed declined levels of self-esteem and increased levels of loneliness, leading to the possible conclusion that it had a harming effect on already troubled individuals [36]. Other research suggests a ‘potential malicious cycle of unregulated Internet use’ which means that those relying too much on the Internet to relieve symptoms of loneliness might in turn simply increase the likelihood of developing additional problems in their lives [32, 37, 38].

In Australia around 112,000 children and adolescents suffer from major depressive disorder, nearly 300,000 or 7.4 % have ADHD, and 10.7 % of adolescent girls and 4.5 % of adolescent boys had seriously considered attempting suicide in the previous year [39]. While these figures alone are cause for concern, it is common for youth already experiencing mental disorders also to have higher rates of engaging in problem behaviours that could put them at risk physically and mentally [40]. These behaviours might include drinking alcohol, smoking, taking illicit substances and sexual intercourse. For example, 27.6 % who have major depressive disorder also binged on alcohol in the previous month, 13 % had used cannabis and one quarter had smoked [39]. Similar to these links with mental disorders, research also suggests associations between this risky behaviour with problematic online behaviour [29, 34, 41–47]. Again, conclusions have not been drawn as to the order in which one affects the other but a pattern is suggested linking mental disorders, risk taking behaviour and problematic online behaviour.

Technology is developing at a rapid rate and it is virtually impossible to deliver up-to-date information with which to devise appropriate treatment strategies for problem behaviours associated with its use. However, given that over a third of young Australians use the Internet to source information about mental health problems [48], there is potential to develop effective online services which can engage with young people at risk and perhaps increase their feelings of connectedness to others. The reliance on using technology to find help and social support may be an important tool for those with mental health issues, given the rates of young people using services for mental health problems remain sub optimal [49].

While a significant body of research into problematic Internet use and its associations with mental disorders exists, few studies appear to have been conducted at the population level internationally [50], and none in Australia [51, 52]. We report here the national estimates of Internet and electronic gaming collected on Australian young people aged 11–17 years in Young Minds Matter. Specifically, we aim to:

1. Present national estimates of the frequency and intensity of Internet use and electronic gaming by Australian children and adolescents; as well as the proportion and characteristics of those children and adolescents who exhibit problematic Internet or electronic gaming behaviour;
2. Describe the co-morbidity of this problematic behaviour with mental disorders; and
3. Describe the co-variation of these estimates with key social and demographic variables; and risk taking behaviours.

This data will contribute a population-wide, contemporary view of problematic Internet use and electronic gaming behaviour by Australian adolescents, which may be of use in designing relevant and timely interventions.

## Methods

### Population

Young Minds Matter was conducted during 2013–14 in respect of children 4–17 years living in Australia. In total, 6,310 parents and carers across Australia participated in the survey, which represented a modest household response rate of 55 %. Data was captured from parents and carers via personal interview, while self-report questionnaires were also completed by 2,967 children aged 11–17 years in these households (representing an 89 % response rate of eligible children) using a tablet computer. Survey methodology has been described in detail elsewhere [7]. Results presented here are confined to children aged 11–17 years.

Through comparison with data from the 2011 Australian Census of Population and Housing, the sample was found to be representative of the normal Australian population distribution of 11–17 year olds, based on socio-economic status; family structure; country of birth, education status and labour force status of parents/carers; housing tenure; and household income. To identify possible patterns of non-response and bias in our data, we conducted small area modelling of response rates, as well as comparing our survey results with those from other surveys, such as the British Child and Adolescent Survey of Mental Health and Wellbeing [53]. As such, we did not find any systematic bias in our response rates. In addition, the Strengths and Difficulties Questionnaire (SDQ) in the YMM measured a prevalence of 10.1 % of 4–17 year-olds in the abnormal range (95 % CI = 9.2–11.0 %), which compared very closely to the prevalence of 10 % based on a normative sample of British children and adolescents from the 1990s. While promising, none of these analyses ensure that our results are entirely without bias.

### Variables

#### Internet and electronic games use

Due to the overall length of the survey, it was not possible to include an extensive set of questions related to use of the Internet and electronic gaming. Electronic game use included games played on a gaming console or online, using a handheld device, a computer, or mobile phone. Internet use excluded time spent on school or work related activities. Separate questions about each activity were asked relating to the frequency and timing of use (Table 1). To measure problem Internet use and electronic gaming behaviour, Young Minds Matter incorporated and adapted a scale drawn from the EU Kids Online Survey [15], which measures behavioural aspects of Internet use by children and enables the identification of those young people exhibiting Internet and electronic gaming behaviour which may be considered problematic. Although this scale is not as widely used as Young’s scale for identifying Internet Addiction [2] or the Chen Internet Addiction Scale [54], it is similar in intent and purpose and can provide a useful tool for diagnosing those children at risk of developing more serious Internet use problems. With YMM data, Cronbach’s alpha was calculated as 0.68. (No similar measure could be located online from the EU Kids Online website.) Again, due to space constraints, questions were asked of the two activities combined to determine if time spent on them interfered with the young person’s normal daily activities. These questions sought information about five specific behaviours that may be indicative of addiction to the Internet, social media or electronic gaming (Table 1).

**Table 1:** Items used in the second National Mental Health Survey of Child and Adolescent Mental Health and Wellbeing to measure behaviour related to Internet use and electronic gaming

The next question(s) are about your internet use. This may be internet accessed on a computer, mobile phone or tablet. Internet use includes accessing social media such as Facebook or Twitter, emailing, looking at websites or chatting online. The questions are about the time you use the internet which is not related to your school work or for work purposes.
YIU3. Do you use the internet?
2 – Yes
0 – No
ASK IF YIU3 = YES.
YIU4. On an average weekday approximately how much time do you spend on the internet?
1 - Less than 1 hour
2 - 1–2 hours
3 - 3–4 hours
4 - 5–6 hours
5 - 7–8 hours
6 - 9–10 hours
7 - 11 hours or more
YIU5. On an average day on the weekend approximately how much time do you spend on the internet?
1 - Less than 1 hour
2 - 1–2 hours
3 - 3–4 hours
4 - 5–6 hours
5 - 7–8 hours
6 - 9–10 hours
7 - 11 hours or more
ASK ALL.
The next question(s) are about your electronic game use. Electronic games can be games that you play on an Xbox or similar console, online, on a handheld device, your computer, or mobile phone.
YIU6. Do you play electronic games?
2 – Yes
0 – No
ASK IF YIU6 = Yes
YIU7. On an average weekday approximately how much time do you spend playing electronic games?
1 - Less than 1 hour
2 - 1–2 hours
3 - 3–4 hours
4 - 5–6 hours
5 - 7–8 hours
6 - 9–10 hours
7 - 11 hours or more
YIU8. On an average weekend day approximately how much time do you spend playing electronic games?
1 - Less than 1 hour
2 - 1–2 hours
3 - 3–4 hours
4 - 5–6 hours
5 - 7–8 hours
6 - 9–10 hours
7 - 11 hours or more
ASK IF YIU3 = YES OR YIU6 = YES.
YIU9. Do you go without eating or sleeping because of the internet or electronic games?
1 - Never/almost never
2 - Not very often
3 - Fairly often
4 - Very often
YIU10. Do you feel bothered when you cannot be on the internet or play electronic games?
1 - Never/almost never
2 - Not very often
3 - Fairly often
4 - Very often
YIU11. Do you catch yourself surfing the internet or playing electronic games when you are not really interested?
1 - Never/almost never
2 - Not very often
3 - Fairly often
4 - Very often
YIU12. Do you spend less time than you should with family or friends or doing school work/work because of the time you spent on the internet or playing electronic games?
1 - Never/almost never
2 - Not very often
3 - Fairly often
4 - Very often
YIU13. Have you tried unsuccessfully to spend less time on the internet or playing electronic games?
1 - Never/almost never
2 - Not very often
3 - Fairly often
4 - Very often

For analysis and reporting purposes, problematic behaviour related to Internet use and electronic gaming was defined as the existence of at least four out of the five behaviours. This is referred to as ‘problem behaviour’ in this paper.

#### Mental disorders

Mental disorders captured via the parent/carer report were assessed using the *Diagnostic Interview Schedule for Children Version IV* (DISC-IV) [55]. The DISC-IV implements the criteria for mental disorders set out in the *Diagnostic and Statistical Manual of Mental Disorders*, 4th edition [56]. For the youth self-report, only one measure on major depressive disorder was included, mainly to reduce respondent burden. Specific disorders examined in this study include major depressive disorder, anxiety disorders, ADHD, and conduct disorder.

Young people also were also asked questions based on the Kessler 10 Psychological Distress Scale (K10) [57] about negative emotional states in the four weeks prior to interview. Their questions included additional Kessler items covering positive mental health, behaviour disorders, and any days when they could not carry out normal activities due to reported distress. For example:

Q. *In the last four weeks, about how often did you feel tired out for no good reason?*
  1. *None of the time*
  2. *A little of the time*
  3. *Some of the time*
  4. *Most of the time*
  5. *All of the time.*

Scores in the ‘very high’ range may indicate a severe mental disorder, while a ‘high’ score may indicate a moderate mental disorder. For YMM, Cronbach’s alpha was calculated as 0.93.

In addition, youth were administered the Strengths and Difficulties Questionnaire (SDQ) [58, 59] in relation to their behaviour during the six months prior to the interview. The SDQ took the form of a series of statements. For example:

Q. *I try to be nice to other people.*
  1. *Not True*
  2. *Somewhat True*
  3. *Certainly True*

Four subscales were examined: emotional problems, conduct problems, hyperactivity, and peer problems. The ratings used were normal, borderline and abnormal. An abnormal SDQ rating is indicative of someone being at substantial risk of clinically significant problems or mental disorders and approximately 10 % of a community sample scores this rating. For YMM data, Cronbach’s alpha was calculated as 0.73.

Although not a mental disorder per se, self-esteem was measured using The Adolescent Self-Esteem Scale (ASQ), a new scale developed for this survey. The scale, which was validated in an adolescent population, consists of 12 items. The scale included both positively and negatively worded items which were answered on a five point Likert scale. For example,

Q. *I am able to stand up for myself and what I believe in.*
  1. * Almost all of the time*
  2. * A lot of the time*
  3. * Some of the time*
  4. * A little of the time*
  5. * Hardly ever*
Q. *I feel useless.*
  1. * Almost all of the time*
  2. * A lot of the time*
  3. * Some of the time*
  4. * A little of the time*
  5. * Hardly ever*

Item scores were summed to determine a total ASQ score. In order to determine a threshold for low self-esteem, ASQ scores were compared to the probability of major depression which impacted on functioning in the previous 12 months. A young person was classified as having low self-esteem if their ASQ score corresponded with a predicted probability of depression which was greater than 0.5. In the YMM data, Cronbach’s alpha was calculated at 0.91 for the scale.

#### Youth risk behaviours and problem behaviours

Questions based on the Youth Risk Behavior Surveillance System (YRBSS) [60] and the Avon Longitudinal Study of Parents and Children [61] were used to capture information on a range of youth risk and problem behaviours. Those included here are: use of tobacco, alcohol, cannabis and other drugs; deliberate self-harm and suicidal behaviour; and sexual behaviour. Content was tailored to be age appropriate, for example, for tobacco and alcohol use, young people aged 11–12 were asked screening questions about ever using these, while young people 13 years and older were asked additional questions about use and amount. Questions about self-harm and suicidal behaviour were limited to young people 12 years and older; while other drug use and sexual behaviour questions were asked of young people 13 years and older.

#### Sociodemographic and other family characteristics

Family type, household income, highest level of parent or carer education, housing tenure, labour force status, and remoteness area were collected using the Australian Bureau of Statistics (ABS) standard formats as far as possible. Remoteness areas were assigned based on the Statistical Area 1 (SA1) of residence at the time of the survey using the ABS Remoteness Areas from the 2011 Census of Population and Housing [62]. In addition, parents/carers were asked if they had ever had a mental health problem as assessed by a doctor or other health professional.

#### Family functioning

A shortened version of the General Functioning Subscale of the McMaster Family Assessment Device was used to classify families into four levels of functioning (Cronbach’s alpha 0.86) [63]. This ranged from very good through to poor, with poor indicating unhealthy family functioning likely to require clinical intervention.

#### Service use

A summary or composite measure of ‘service use’ was created that included all health, school and telephone counselling services, and online services which provided personalised assistance [49].

A full version of the questions asked and the associated response categories are available at www.youngmindsmatter.org.au [64].

### Analysis

The populations presented in this paper are unweighted and relate to a representative sample of 11–17 year-olds in the Australian population. The proportions are weighted to represent 11–17 year olds. Additional sample was included for 16–17 year olds, with the weighting taking this into account.

Univariate and Multivariate models were used to assess the associations between a number of variables and prevalence of four out of five problem behaviours related to Internet/electronic gaming (i.e. problem behaviour).

Multivariate logistic regression analysis was used to determine associations. The Multivariate model included factors that had a Univariate association with problem behaviour and was adjusted for age, sex and geographic level of remoteness. Separate models were created for measures which were all linked to the measurement of psychological distress and might be deemed to be overlapping, that is, K10, SDQ and parent-reported DISC-IV disorders in order to determine their individual level of significance. Those that were not independently significantly associated with problem behaviour, such as psychological distress as measured by SDQ, parent-reported mental disorders, and youth-reported major depressive disorder, were then eliminated in order to produce the most parsimonious model. The final model included suicide attempt in the previous 12 months, current alcohol use, family functioning, and levels of psychological distress (measured by K10). All analysis was undertaken using SAS Version 9.4 [65].

## Results

Unless otherwise stated, results are reported for children aged 11 to 17 years. Results for risk behaviours including self-harm and suicidal behaviour are reported for children aged 12 to 17 years; results for all other risk behaviours are reported for adolescents aged 13–17 years.

### Internet use

Internet use was extremely high at 98.9 % (95 % Confidence Interval (CI) = 98.5–99.3 %) of young people reporting current Internet use, with similar proportions and patterns of activity for males and females. There was slightly lower weekday use compared with weekend use (Table 2).

**Table 2:** Average time spent on the Internet by 11–17 year olds

Average daily time		Internet use on weekdays		Internet use on weekends
	*N*	Per cent (95 % CI)	*N*	Per cent (95 % CI)
Doesn’t use	26	1.1 (0.7–1.5)	26	1.1 (0.7–1.5)
1–2 hours	1248	46.2 (44.1–48.3)	1015	37.2 (35.3–39.1)
3–4 hours	780	24.9 (23.2–26.6)	758	25.9 (24.2–27.6)
5–8 hours	575	17.6 (16.1–19.1)	778	23.7 (22.0–25.4)
9 hours or more	338	10.3 (9.1–11.4)	390	12.1 (10.8–13.4)
Total	2967	100.0	2967	100.0

*CI* Confidence interval

### Electronic games use

**Fig. 1:**
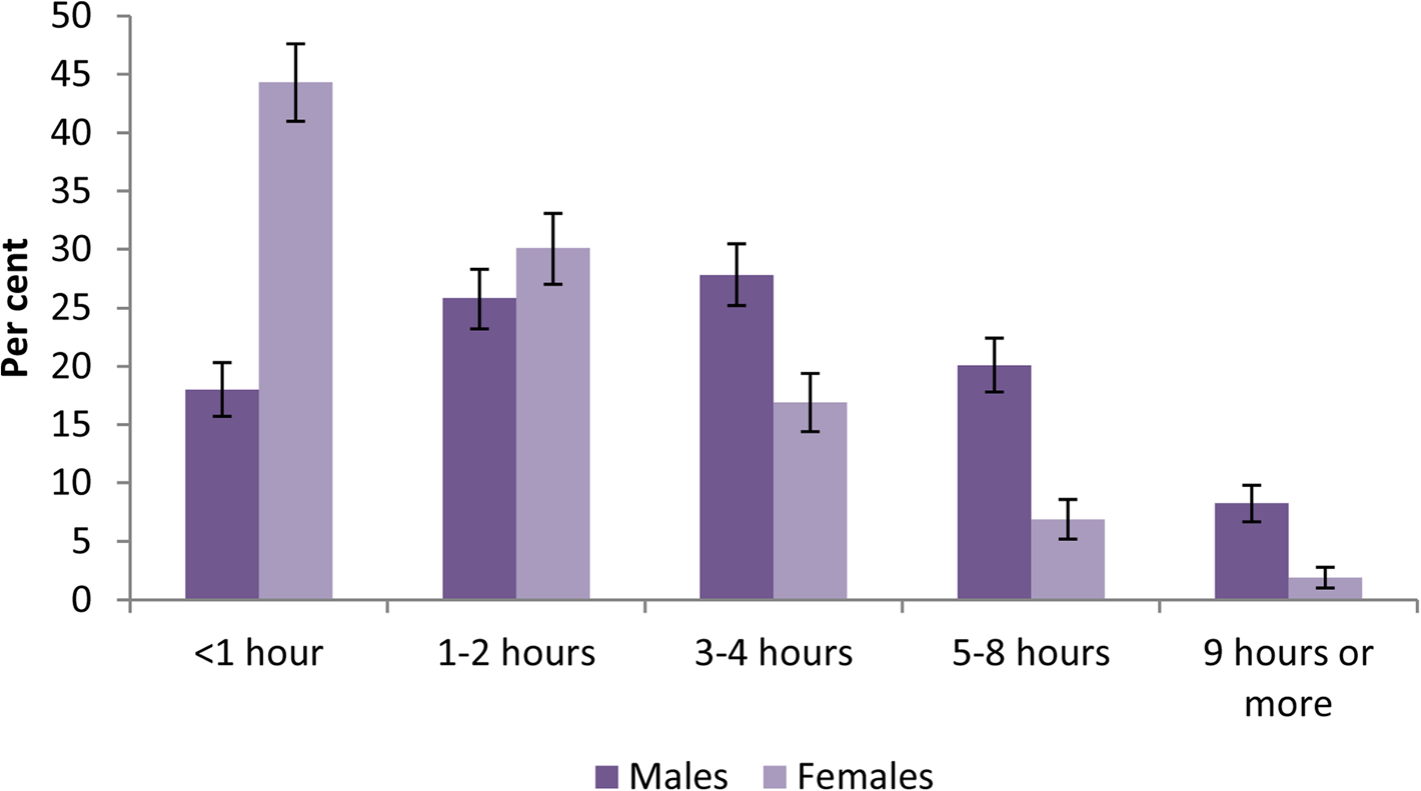
Average daily time spent gaming on weekends by sex. (Excluding those who do not use electronic games.)

In comparison, 2530 or 85.3 % (95 % CI = 83.9–86.6 %) of children aged 11–17 years played electronic games. Only 5.3 % (95 % CI = 4.1–6.5 %) of males did not play electronic games compared with 24.8 % (95 % CI = 22.4–27.2 %) of females. The largest proportion or 24.9 % (95 % CI = 23.2–26.7 %) of children played for less than one hour a day on the weekends, although there was a gender difference in weekend average daily gaming which saw females spending less time gaming than males (Fig. 1).

### Problem behaviours linked to use of Internet or electronic games

The survey identified that 127 or 3.9 % (95 % CI = 3.2–4.6 %) of children had at least four out of five problem behaviours (i.e. problem behaviour) associated with Internet use/electronic gaming (Table 3). Of these, 16 year old females had the highest rate of incidence (8.0 % of all girls, 95 % CI = 5.2–10.9 %), while 15 year old boys had the highest rate for males at 6.4 % (95 % CI = 2.6–10.3 %). Only 29 or 0.9 % (95 % CI = 0.5–1.3 %) of children reported experiencing all five behaviours.

**Table 3:** Prevalence of problem behaviours for children aged 11–17 years

Behaviour	*N*	Per cent (95 % CI)
Went without eating or sleeping	190	5.9 (5.0–6.8)
Feel bothered when not doing	615	20.7 (18.9–22.4)
Use when not really interested	1014	32.0 (30.1–33.9)
Spent less time than should with family or friends, doing school work or work	685	21.6 (20.0–23.1)
Tried unsuccessfully to spend less time	662	23.5 (21.7–25.2)
Problem Internet/electronic gaming behaviour^a^	127	3.9 (3.2–4.6)

*CI* Confidence interval

^a^Four to five behaviours experienced

**Fig. 2:**
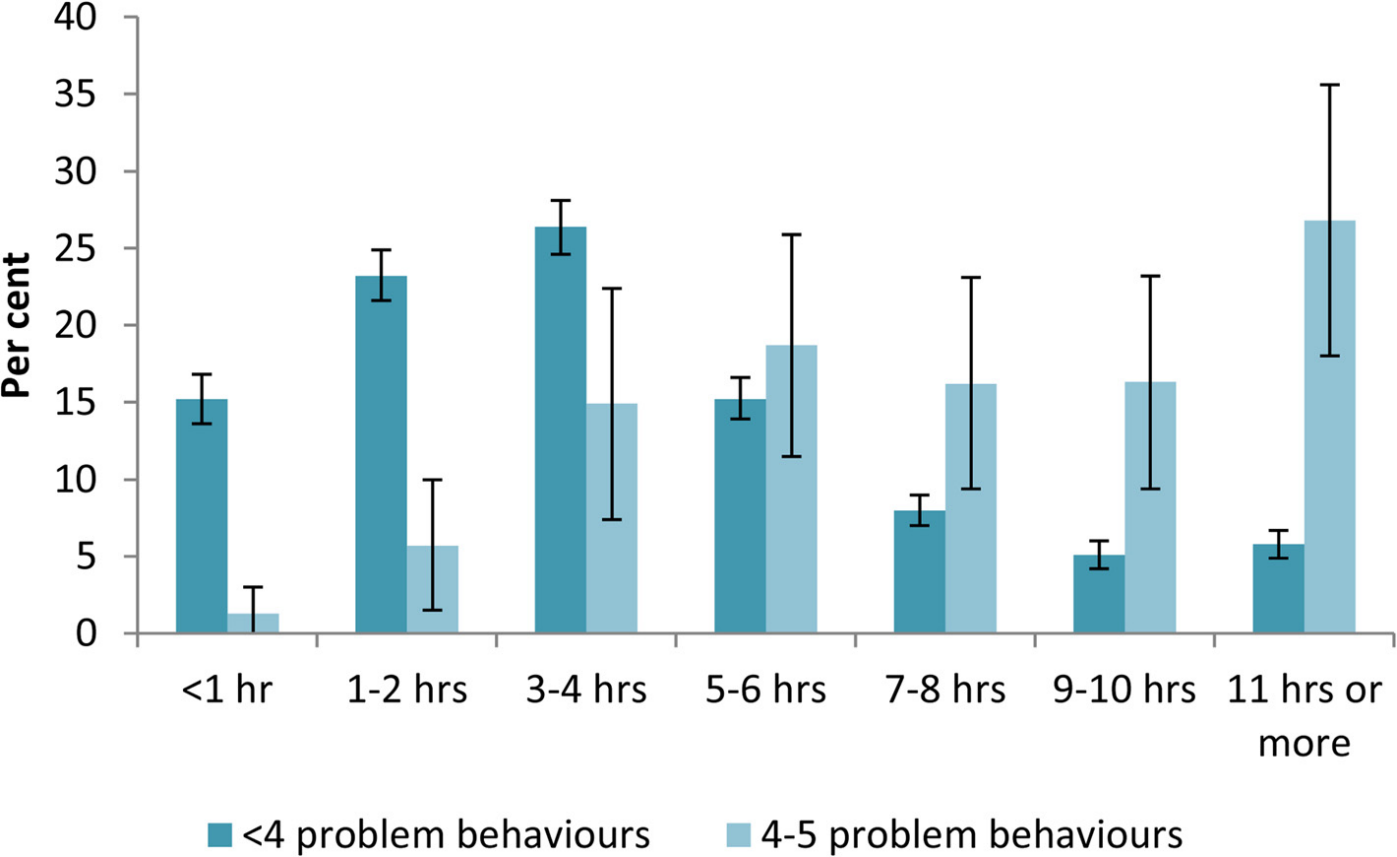
Average daily weekend Internet use by level of problem behaviours

The greatest proportion of children without problem behaviour spent an average of three to four hours a day online during the weekend, compared to the 11 hours or more a day which was the highest category for children with problem behaviour (Fig. 2).

**Fig. 3:**
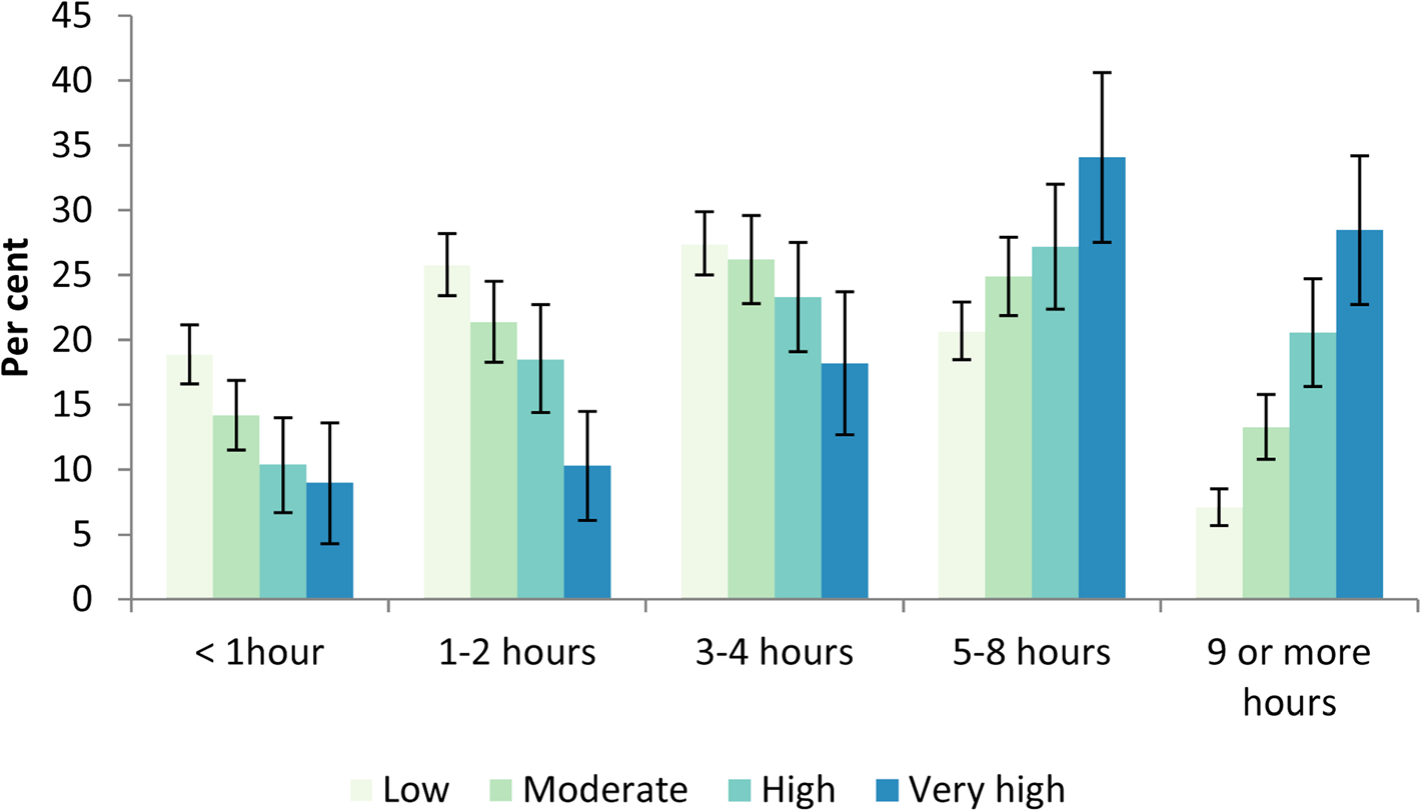
Weekend daily Internet use by K10 category

With the K10 scores, there was a much a higher proportion of children with a very high K10 score (18.2 %, 95 % CI = 12.3–24.0 %), compared to those with a low K10 score (1.5 %, 95 % CI = 0.8–2.2 %) who exhibited problem behaviour. A similar pattern emerged for average daily weekend time spent online, where those scoring in the very high K10 category spent considerably more time on the Internet than those with a low score (Fig. 3). For females with problem behaviour, the proportion with a very high K10 score (41.8 %, 95 % CI = 28.8–54.9 %) was more than twice as high as the proportion for males at 19.4 % (95 % CI = 7.7–31.1 %). (Additional file 1: Table S1.)

For youth with problem behaviour, a little over a third of both females and males recorded abnormal SDQ total difficulties scores compared with only 11 % (95 % CI = 9.2–12.8 %) and 7.4 % (95 % CI = 6.0–8.8 %) respectively for those with fewer or no problem behaviours. (Additional file 1: Table S2.) While the differences in proportions varied, this pattern held true for all problems scored, that is, peer relations problems, hyperactivity, conduct problems and emotional problems.

Univariate analysis showed that socio-demographic associations with problem behaviour were not significant but were indicative of some patterns. The categories which had the highest prevalence of problem behaviour are listed in Table 4. In addition, we found negligible difference in problem behaviour prevalence between children whose parent/carer had ever been diagnosed with a mental problem (4.4 %, 95 % CI = 3.2–5.5 %) and those who had not (3.5 %, 95 % CI = 2.6–4.4 %). See Additional file 1: Table S3 for prevalence rates for all categories of each characteristic.

**Table 4:** Prevalence of problem behaviour for children aged 11–17 years for selected socio–demographic characteristics

Socio–demographic characteristic	*N*	Per cent (95 % CI)
Age group –		
11–15 years	53	3.3 (2.4–4.2)
16–17 years	74	5.5 (4.3–6.7)
Highest level of parent/carer education –		
Bachelor degree or higher	54	4.7 (3.4–6.0)
Diploma or cert III/IV	44	3.0 (2.1–4.0)
Year 11 or 12	13	3.4 (1.5–5.3)
Year 10 or below	16	5.7 (2.7–8.6)
Parent/carer labour force status –		
Both carers employed	59	3.4 (2.5–4.3)
One carer employed, one carer not in employment	29	4.8 (2.8–6.8)
Both carers not in employment	7	8.7 (2.3–15.0)
Sole carer employed	24	3.9 (2.3–5.5)
Sole carer not in employment	8	3.5 (0.9–6.1)
Not stated	0	–
Housing tenure –		
Owned outright	12	2.5 (1.0–4.1)
Owned with a mortgage	65	3.5 (2.6–4.4)
Rented – public housing	9	6.5 (2.4–10.6)
Rented – other	41	5.3 (3.5–7.1)
Other	0	–
Annual household income –		
Less than $52,000	32	4.4 (2.8–6.0)
$52,000–129,000	54	3.6 (2.6–4.6)
$130,000 or more	37	4.3 (2.9–5.8)
Child’s country of birth –		
Australia	107	3.7 (3.0–4.5)
Overseas	20	5.0 (2.6–7.4)
Family type –		
Intact family	73	3.8 (2.9–4.7)
Step family	7	3.7 (0.7–6.7)
Blended family^a^	11	4.4 (1.6–7.1)
Lone parent family	33	3.9 (2.5–5.3)
Other family	3	8.8 (0.00–18.8)
Geographic level of remoteness –		
Major cities	97	4.6 (3.6–5.5)
Inner regional Australia	22	2.8 (1.6–4.0)
Outer regional Australia	6	2.6 (0.2–5.0)
Remote Australia	2	3.7 (0.0–8.8)

*CI* Confidence interval

^a^Blended families include those with 2 or more children, at least one of whom is the natural or adopted child of both parents, and at least one who is the step child of one of them

Further Univariate analysis showed that young people engaging with a range of risk factors reported higher prevalence of problem behaviour than their counterparts (Table 5). For example, around one in four young people who had attempted suicide in the previous 12 months exhibited problem behaviour, compared with only one in 29 of those who had not attempted suicide. Additional file 1: Table S4 shows similar results but from the perspective of whether or not the child had problem behaviour. Those with problem behaviour had a much higher rate of risk prevalence.

**Table 5:** Prevalence of problem behaviour by presence of risk factors^a^

Risk factors	Proportion with risk factor	Whether problem behaviour by presence of risk factor			
		No		Yes	
	Per cent (95 % CI)	*N*	Per cent (95 % CI)	*N*	Per cent (95 % CI)
K10 score –					
Low	50.9 (49.0–52.8)	1430	98.5 (97.8–99.2)	23	1.5 (0.8–2.2)
Moderate	29.1 (27.3–31.0)	836	96.6 (95.4–97.8)	33	3.4 (2.2–4.6)
High	13.3 (12.0–14.7)	385	92.9 (90.2–95.6)	32	7.1 (4.4–9.8)
Very high	6.6 (5.7–7.6)	189	81.8 (76.0–87.7)	39	18.2 (12.3–24.0)
Major depressive disorder^b^	7.6 (6.7–8.6)	92	3.1 (2.4–3.8)	35	13.0 (8.6–17.4)
Self–harmed	6.9 (5.9–7.8)	96	3.2 (2.5–3.9)	31	13.6 (8.7–18.5)
Attempted suicide^c^	2.1 (1.6–2.6)	110	3.4 (2.8–4.1)	17	26.5 (14.7–38.3)
Current alcohol user	13.0 (11.7–14.2)	71	4.0 (3.1–5.0)	42	7.8 (5.4–10.2)
Binged on alcohol	9.0 (8.0–10.0)	81	4.2 (3.3–5.1)	32	8.3 (5.5–11.2)
Current smoker	5.1 (4.3–6.0)	96	4.4 (3.5–5.3)	17	8.6 (4.5–12.8)
Ever used cannabis	11.6 (10.2–13.0)	80	4.0 (3.1–4.9)	33	10.1 (6.7–13.5)
Other drug user^d^	4.5 (3.6–5.4)	99	4.4 (3.5–5.3)	14	10.7 (5.0–16.5)
Ever had sex	14.9 (13.4–16.4)	80	4.3 (3.3–5.2)	33	7.3 (4.8–9.8)
Low self–esteem^e^	2.4 (1.9–3.0)	112	3.5 (2.8–4.2)	15	19.9 (10.1–29.6)
Used services^f^	18.0 (16.2–19.7)	71	3.6 (2.8–4.5)	42	9.6 (6.6–12.5)
Family functioning-Poor	4.1 (3.3–5.0)	119	93.3 (88.8–97.8)	10	6.7 (2.2–11.2)
Very good	57.9 (55.8–60.0)	1620	96.7 (95.8–97.6)	60	3.3 (2.4–4.2)

*CI* Confidence Interval

^a^Age ranges for risk factors: 11–17 yrs – K10, major depressive disorder, self–esteem, family functioning; 12–17 yrs – self–harm, suicide attempt; 13–17 yrs – current alcohol user, binged on alcohol, current smoker, ever used cannabis, other drug user, ever had sex, used services

^b^Youth–reported

^c^In previous 12 months

^d^Includes using prescription drugs for non–medical purposes; ecstasy; amphetamines and methamphetamines; cocaine; hallucinogens such as LSD; inhalants such as petrol, glue, aerosols, paint, solvents or nitrous; heroin; steroids; GHB or ketamine

^e^As measured by DISC IV and equating to a 50 % probability of having depression causing an impact on functioning in the previous 12 months

^f^Youth–reported use of any service for emotional or behavioural problems in the past 12 months

In multivariate logistic regression, several variables were associated with problem behaviour (Table 6). For example, children who had attempted suicide in the previous 12 months were three times more likely to have problem behaviour and those who suffered from very high levels of psychological distress were more than nine times as likely to have problem behaviour.

**Table 6:** Odds ratios for selected variables and problem behaviour for adolescents aged 13–17 years

Variable	Univariate OR^a^ (95 % CI)	Multivariate OR^b^ (95 % CI)
Attempted suicide^c^	7.5 (4.2–13.6)	3.0 (1.5–6.2)
Current alcohol use	2.1 (1.5–3.2)	1.4 (0.9–2.3)
Family functioning –		
Fair vs very good	1.8 (1.1–2.9)	1.5 (0.9–2.7)
Poor vs very good	2.3 (1.2–4.4)	1.4 (0.6–3.0)
Good vs very good	1.2 (0.8–1.9)	1.1 (0.6–1.8)
K10 categories –		
Moderate vs low	2.5 (1.4–4.2)	2.2 (1.2–4.2)
High vs low	5.2 (2.9–9.2)	4.6 (2.3–9.2)
Very high vs low	12.9 (7.3–22.8)	9.6 (4.7–20.1)
SDQ categories^d^ –		
Abnormal vs normal	7.1 (4.7–10.8)	
Borderline vs normal	3.1 (1.9–5.0)	
Parent–reported DISC IV disorder^e^	2.2 (1.5–3.2)	
Youth–reported major depressive disorder	4.2 (2.8–6.3)	

*CI* Confidence Interval

^a^Odds ratio from a separate logistic regression model for each characteristic, only adjusting for age, sex and that characteristic;

^b^Odds ratio from overall multivariate logistic regression model including all characteristics in model and age and sex and remoteness;

^c^In previous 12 months

^d^Characteristic not significantly associated with prevalence of problem behaviours in final multivariate model

^e^Includes social phobia, separation anxiety disorder, generalised anxiety disorder, obsessive–compulsive disorder, major depressive disorder, ADHD, and conduct disorder

## Discussion

The intent of this paper was to report on current Internet use and electronic gaming behaviour by 11–17 year old Australians, with specific focus on problematic online behaviour and its associations with diagnosed mental disorders, risk taking behaviour and socio-economic characteristics. Some 3.9 % of children and adolescents were found to experience problematic Internet or electronic gaming behaviour. Although International comparisons are plagued by lack of definitional and measurement consistency, this level of prevalence is in keeping with other studies, which indicate that Asian countries seem to suffer from higher rates of problematic Internet behaviour than do Western countries (e.g. 0.8 % using Young’s Internet Addiction test in Italy compared to 20.3 % in South Korea) [24].

A small but important number of associations were found which are of concern. Having attempted suicide in the previous 12 months, suffering from high levels of psychological distress, and consuming alcohol were all associated with problem behaviour. Due to the cross-sectional nature of this study, we were unable to determine the direction of these associations and the potential mediating effect of these variables on problem behaviour, however, our findings were similar to other research which found associations with IA or excessive Internet use and psychological distress [66], depression [2–4, 18, 67], suicide ideation [28, 68], self-harm [69], and alcohol abuse [41, 42, 47]. From this we can deduce that Australian adolescents show patterns of comorbidity which mostly mirror those from elsewhere. Even though rates of problem Internet use and electronic gaming behaviour appear relatively low, the links between this behaviour and other mental health problems and risk taking behaviour signal the need for closer scrutiny.

Although a wide range of socio-demographic and other variables were also tested in the models, only fair and poor family functioning showed any association with problem behaviour. A pattern, albeit weak, also emerged indicating that children from households with carers not in employment, in a blended family environment and living in public housing have relatively higher prevalences of problem behaviour. This could partly be explained by the need for those children to escape from their immediate environments into the cyber world, where support can be found either through chat rooms, social media or through multi-player gaming environments. There is a wide body of literature which supports links between IA and dysfunctional family background [29, 34, 46, 70]. Studies have found that lower or poor family function and parenting styles lacking in emotional warmth were predictors for Internet addiction [71, 72], thus illustrating the importance of adopting a family-based preventive approach which takes account of intra-family conflict [72].

After accounting for psychological distress, we found no independent associations between any of the parent/carer or youth-reported disorders or youth-reported self-esteem and problem behaviour. However, the survey also included the Strengths and Difficulties Questionnaire (SDQ), which captured information on several subscales - emotional problems, conduct problems, hyperactivity, and peer problems. While links were found between each subscale and problem behaviour, the most compelling was found with the emotional problems subscale, with nearly half of females and a quarter of males with problem behaviour scoring an abnormal rating for emotional problems. Taken together with the strong association with high levels of psychological distress as measured by the K10, this suggests a plausible link between dysphoric youth and problem behaviour, which is in keeping with other research [29, 70]. Ha et al. [3] found that depressive symptoms were a significant predictor for Internet addiction (IA) and that online activity may take the form of self-soothing behaviour in reaction to a depressive mood, especially for those with internalization tendencies. According to Young “an Internet addict’s use of the computer is less about using it for information and more about finding a psychological escape to cope with life’s problems” [2]. Davis [31] proposed a theory that psychopathologies such as depression can pre-exist a condition he characterizes as Pathological Internet Use (PIU), while Dong [30] used a longitudinal study to identify depression and anxiety as pathological traits which may trigger Internet addiction. Koo et al. [73] postulated that younger people are more likely to become addicted to the Internet due to psychosocial distress or what they characterize as intrapersonal conditions, such as a desire to ‘escape from self’ and low self-control, and they propose that preventative therapies targeted at their intrapersonal vulnerabilities may be useful also in treating their Internet addiction. In should be kept in mind, however, that Davis also pointed out that the symptoms of PIU may differ markedly from those of depression and thus both conditions must be treated separately [31]. Treating one will not necessarily fix the other.

For all of the risk factors reported in this study, children exhibiting the risk factor always had a higher prevalence of problem behaviour. This result replicates other research which found that a wide variety of risk behaviours were associated with problematic Internet behaviour [29, 45, 74]. However, there were some differences in our results compared with findings from other studies. Researchers have identified a link between IA and ADHD [75–77] and mental health problems in parents/carers [76]; however, we did not find either of these variables linked with problem Internet/electronic gaming behaviour, although Univariate analysis did show that boys suffering from hyperactivity had higher levels of problem behaviour. YMM was a population based study, with a representative sample of adolescents, therefore, the results must be accepted as true in the Australian context and it is possible that cultural anomalies or methodological differences may explain differences in results between studies.

When measured against the effects of psychological distress, we also did not identify strong associations between problem behaviour and socio-demographic variables such as household income, tenure type, remoteness area, or family type, which indicates that problem behaviour may be widely dispersed throughout the community, especially where it co-exists with high levels of distress.

Time use of the Internet is not a direct function in diagnosing Internet addiction; however, studies have shown that addictive behaviour can be associated with increased online activity [2, 67, 78]. Moreover, Ybarra et al. [79] found that depressive symptomatology in adolescents was associated with longer (or more intense) Internet use, as opposed to frequency of use. A pattern emerged of excessive online activity captured through the survey which may indicate links between this behaviour and other problems. Not surprisingly the proportion of children with problem behaviour who spent nine hours or more a day online during the weekend was four times higher than for others. The pattern was similar for children with very high compared to low levels of psychological distress (the proportion for very high was four times higher than for low). Davis [31] proposed in his cognitive-behavioral model of pathological Internet use (PIU) that it is usual for PIU sufferers to spend more and more time on the Internet, to the extent that they isolate themselves from friends and family in favour of online friends and online experiences. This in turn leads to feelings of guilt and isolation which exacerbates the condition. Aa et al. [80] found that compulsive Internet use was more likely to be linked to loneliness for adolescents who were characterized as introverted, low-agreeable and emotionally less-stable. Moves to limit screen time for children outside of study purposes are gaining traction; however, simply abbreviating screen based activity may overlook an underlying mental health condition and may be an over-simplified approach to the wrong problem. Parents should be encouraged to understand their child’s online activity in terms of both its content and its intent.

Unlike others who have found that males are more likely to be Internet addicts than females [18, 41, 78, 80–86], this was not the case with Young Minds Matter, which found little difference between the sexes with respect to problem behaviour itself. Heo [83] postulates that high expectations of academic achievement for boys in Asian cultures may motivate them to ‘hide out from reality’ through Internet activity, relieving stress but leading to poor academic achievement which in turn compounds the problem. It is possible that motivators for Australian adolescents to spend more time online may be different. A review of epidemiological studies of Internet Addiction found both genders to be associated with the condition and the paper cited cultural differences, as well as the different measures used, as possible explanations for contradictory findings between countries [50]. Moreover, a recent Australian report [87] showed that teenage girls aged 14–17 years were more likely to have gone online than their male counterparts (85 % compared to 78 %), which may be a recent phenomenon in Australia indicating a shift in gendered online activity patterns. Indeed, gender differences in YMM were apparent in patterns of online activity and also in the associations between other problems and problem behaviour. Although both genders reported spending equal amounts of time online, boys were more likely than girls to be electronic gamers, with the literature supporting this finding [84, 88–90]. For girls, we observed a psychopathological trend showing an association between very high K10 scores, emotional problems and prevalence of problem behaviour. In all instances, their rates were at least double that for boys. A possible explanation is that females suffering from mental disorders are more likely to internalize their emotions [91] and thus find solace in the cyber world, which they tend to use for purposes of communication and socialization, unlike males who prefer to go online to seek entertainment (typically gaming or videos) and information [74, 82, 91, 92]. Ko et al. [85] found depression and social phobia in girls to be a predictor of Internet addiction, and this links with the findings that adolescent Australian females suffer depression at twice the rate of their male counterparts [93]. In contrast, YMM showed that boys with problem behaviour were more likely than girls to suffer from conduct or hyperactivity problems – echoing studies which show hostility and ADHD in boys as risk factors for Internet addiction [73, 85].

Taken in its totality, existing literature on gender differences in Internet use and its effects on psychological health are inconclusive, thus leaving open the question yet again about cause and effect where the Internet and mental health are concerned. Nevertheless, in determining treatment regimens for adolescents with either mental disorders or problem Internet use and electronic gaming behaviour, the literature and our findings illustrate the importance of being aware of gender differences and developing protective behaviours appropriately. For example, Muller’s findings [94] that Internet gaming disorder in boys is associated with decreased conscientiousness and a tendency to compete rather than cooperate may indicate that moderating these behaviours in boys may go some way in assisting them to break or avoid dependence on electronic gaming. In contrast, providing girls with tools to assist them in dealing with, or preventing, high levels of psychological and emotional distress would be of greater benefit.

### Limitations

The study was cross-sectional, therefore causality cannot be assumed and the direction of the associations is unknown. For example, we are unable to determine how all of the variables affect each other and whether or not factors such as poor family functioning or parental mental health problems are influenced by the child’s problem behaviour or vice versa, that is, if they are mediating or confounding variables. While this may have some implications for the validity of our findings, this level of analysis was not the focus of the paper. Further research would be needed to investigate these associations in greater detail.

We cannot know if young people in non-participating families would be more or less likely to have had mental disorders or problem behaviour compared to those in our survey. Every effort was made to ensure the sample obtained was representative of the general population.

Information was obtained via self-report from young people whose judgment and reporting of their behaviour may be inconsistent – what may be judged as excessive by one may not be by another. Although the DISC applies standard DSM-IV diagnostic criteria and is a well-validated tool with good concordance with clinical diagnoses [95], our results relied on information provided by young people themselves to determine diagnostic status. No information was collected on the nature of the Internet use or the type of electronic gaming activity.

The literature mostly discusses electronic gaming and Internet use as separate activities; however, due to space and timing restrictions, the survey was unable to capture information about electronic gaming in isolation from Internet use and the two variables have been combined. Therefore, it is not possible for us to speculate on whether problem behaviour stems from electronic gaming, Internet use or both. As a result, these survey findings are indicative of problems with screen based activity (excluding TV viewing) in general. While this is unfortunate, Kuss [50] notes that Internet use and online gaming have also been conflated in the DSM-5 in the diagnostic criteria for Internet Gaming Disorder. The same paper also noted that the use of online gaming was associated with Internet addiction in adolescents [50]. From a behavioural perspective, Peng et al. [96] found that online gaming was associated with maladaptive cognitions, shyness and depression – all variables shown to contribute to Internet dependency. This may ameliorate this particular limitation to our study.

Lastly, we decided to define problem behaviour as exhibiting at least four out of five behaviours due to our relatively small sample size compared with the EU study where the scale originated. This means that our results are not directly comparable with those of other studies using this scale, which define problem behaviour as exhibiting five out of five behaviours.

## Conclusion

The pervasiveness of new technology in every facet of young people’s lives has led to a change in cultural ecology and the traditional ways in which they may deal with issues of social isolation, bullying, depression, behavioural disorders, boredom, or family breakdown. Quite often they will turn to a screen instead of another person, with a third of them sourcing information online about mental health problems [48] and many using the Internet to remain socially connected. While we found disturbing links between problem behaviour in young people and a number of mental disorders and risky behaviours, this study cannot determine whether the latter are precursors or sequelae. Regardless, these associations are cause for concern amongst parents, educators and service providers, particularly with respect to links identified between youth suicide attempts, high levels of psychological distress, and problem behaviour.

While Young Minds Matter provides a unique and important contribution to the field, its cross-sectional nature means that prospective studies would be useful to help tease out the chicken and egg nature of problem behaviour as defined by this study and the accompanying dysphoric behaviours. Longitudinal studies designed to explore the potential mediating roles played by factors such as psychological distress, poor family functioning and alcohol consumption would assist in the development of appropriate treatment programs for youth presenting with problem behaviour. A key issue identified by other research [45, 46, 97] is to take account of all problems presented by any individual as a group, rather than simply to treat each in isolation, while ignoring how each interacts with the other. In the United States it has also been noted that as patients usually present for the conditions which are comorbid for Internet addiction, there is a risk the Internet addiction itself may be missed unless it is specifically looked for [22]. Therefore, it is important for local practitioners to develop interventions for problem behaviour which are mindful of the associations identified in this study, particularly where they are gender-related, and for them to consider the full mosaic of possible contributors. A strong focus should be given to making more online programs available, which target young people suffering from suicide ideation and psychological distress, who may turn to the Internet for solutions to their internal struggles. Ideally, parents should also acquaint themselves with their children’s online activity and explore why their child may be unable to disconnect from the virtual world. Efforts by parents to enhance their child’s emotional regulatory abilities in adolescence may go some way towards reducing or avoiding addiction to the Internet [98].

## Ethics approval

The survey was conducted with the approval of UWA’s Human Research Ethics Office. The survey also received ethics approval from the (then) Australian Government Department of Health and Ageing Human Research Ethics Committee (reference no. 17/2012). Written consent to participate was obtained from the parent/carer of the child and from all young people aged 11–17 years who completed a self-report questionnaire.

## Consent for publication

Not applicable.

## Availability of data and materials

Due to confidentiality restrictions related to the ethics approval for this study, no identifying information about participants may be released. Therefore, the full dataset supporting the conclusions of this article is available to researchers via the Australian Data Archive, http://www.ada.edu.au. The dataset is in the form of a Confidentialised Unit Record File and access conditions apply, requiring any request to be accompanied by an ethics approval, which ensures the confidentiality of all survey participants. The dataset is accompanied by a Technical Manual which contains source codes for the data. A wide range of resources related to Young Minds Matter is also available from http://www.youngmindsmatter.org.au. These resources include detailed tables of data (accessed via the Survey Results Query Tool), the Survey User’s Guide, the Data Access Statement, and copies of all questionnaires and materials used in the survey.

## Additional file

Additional file 1: Table S1. Problem internet/electronic gaming behaviour problems by Kessler10. **Table S2.** Problem internet/electronic gaming behaviour by strengths and difficulties (SDQ) measures by sex. **Table S3.** Prevalence of 4–5 problem behaviours for selected socio-demographic characteristics for children 11–17 years. **Table S4.** Prevalence of risk factors by level of problem behaviours for children and adolescents^1^. (DOCX 23 kb)

## Abbreviations

ABS: Australian Bureau of Statistics
ADHD: Attention Deficit and Hyperactivity Disorder
ASQ: Adolescent Self-esteem Scale
AU: Australian
Cert III/IV: Certificate III/IV
CI: Confidence Interval
DISC-IV or DISC: Diagnostic Interview Schedule for Children Version
DSM-5: Diagnostic and Statistical Manual of Mental Disorders
EU: European
GHD: Gamma hydroxybutyrate
IA: Internet Addiction
K10: Kessler 10 Psychological Distress Scale
LSD: Lysergic acid diethylamide
N: Number
OR: Odds ratio
PIU: Pathological Internet Use
SA1: Statistical Area 1
SDQ: Strengths and Difficulties Questionnaire
UWA: The University of Western Australia
YMM: Young Minds Matter
YRBSS: Youth Risk Behavior Surveillance System
