# Supplementary material for: Internet use and electronic gaming by children and adolescents with emotional and behavioural problems in Australia – results from the second Child and Adolescent Survey of Mental Health and Wellbeing
Source: BMC Public Health. 2016 May 13;16:399. doi: 10.1186/s12889-016-3058-1 (PMC4866411; doi:10.1186/s12889-016-3058-1)
Supplement: Additional file 1: Table S1. — Problem internet/electronic gaming behaviour problems by Kessler10. Table S2. Problem internet/electronic gaming behaviour by strengths and difficulties (SDQ) measures by sex. Table S3. Prevalence of 4–5 problem behaviours for selected socio-demographic characteristics for children 11–17 years. Table S4. Prevalence of risk factors by level of problem behaviours for children and adolescents1. (DOCX 23 kb) [file 12889_2016_3058_MOESM1_ESM.docx]

# Additional file 1

| Table S1. Problem internet/electronic gaming behaviour problems by Kessler10 | | | |
| --- | --- | --- | --- |
|  |  | Females | Males |
|  |  | Per cent (95% CI) | Per cent (95% CI) |
| 4-5 problem behaviours | **K10 categories** |  |  |
| No | Low | 46.4 (43.4-49.4) | 57.6 (55.0-60.2) |
|  | Moderate | 29.5 (26.7-32.3) | 29.1 (26.6-31.6) |
|  | High | 16.0 (14.0-18.0) | 9.9 (8.2-11.7) |
|  | Very high | 8.1 (6.7-9.5) | 3.3 (2.4-4.3) |
|  | Total | 100.0 | 100.0 |
| Yes | Low | 15.6 (5.2-25.9) | 24.1 (11.6-36.6) |
|  | Moderate | 16.8 (7.0-26.7) | 34.0 (20.8-47.1) |
|  | High | 25.8 (14.6-37.0) | 22.5 (9.9-35.1) |
|  | Very high | 41.8 (28.6-54.9) | 19.4 (7.7-31.1) |
|  | Total | 100.0 | 100.0 |

CI – Confidence interval

| Table S2. Problem internet/electronic gaming behaviour by strengths and difficulties (SDQ) measures by sex | | | |
| --- | --- | --- | --- |
|  |  | **Females** | **Males** |
|  |  | **Per cent (95% CI)** | **Per cent (95% CI)** |
| 4-5 problem behaviours | **Total difficulties score** |  |  |
| No | Normal | 75.5 (72.9-78.1) | 79.9 (77.7-82.0) |
|  | Borderline | 13.5 (11.5-15.5) | 12.7 (10.9-14.6) |
|  | Abnormal | 11.0 (9.2-12.8) | 7.4 (6.0-8.8) |
|  | Total | 100.0 | 100.0 |
| Yes | Normal | 39.3 (26.5-52.1) | 42.3 (28.7-56.0) |
|  | Borderline | 22.4 (11.3-33.5) | 25.9 (13.5-38.3) |
|  | Abnormal | 38.4 (25.9-50.8) | 31.8 (19.0-44.6) |
|  | Total | 100.0 | 100.0 |
| 4-5 problem behaviours | **Peer relations problems** |  |  |
| No | Normal | 78.7 (76.2-81.1) | 80.9 (78.7-83.0) |
|  | Borderline | 15.9 (13.8-18.0) | 15.5 (13.5-17.5) |
|  | Abnormal | 5.5 (4.2-6.8) | 3.6 (2.6-4.7) |
|  | Total | 100.0 | 100.0 |
| Yes | Normal | 63.7 (51.4-76.1) | 68.6 (55.6-81.6) |
|  | Borderline | 20.8 (10.6-31.0) | 24.7 (12.6-36.9) |
|  | Abnormal | 15.5 (6.0-25.0) | 6.6 (0.4-12.9) |
|  | Total | 100.0 | 100.0 |
| 4-5 problem behaviours | **Hyperactivity** |  |  |
| No | Normal | 78.3 (75.9-80.6) | 75.2 (72.9-77.7) |
|  | Borderline | 9.8 (8.1-11.4) | 11.3 (9.5-13.0) |
|  | Abnormal | 12.0 (10.1-13.8) | 13.5 (11.6-15.4) |
|  | Total | 100.0 | 100.0 |
| Yes | Normal | 53.5 (40.4-66.5) | 36.1 (22.1-50.1) |
|  | Borderline | 12.8 (5.8-19.7) | 18.3 (8.3-28.4) |
|  | Abnormal | 33.8 (21.7-45.8) | 45.6 (31.9-59.2) |
|  | Total | 100.0 | 100.0 |
| 4-5 problem behaviours | **Conduct problems** |  |  |
| No | Normal | 87.4 (85.4-89.4) | 81.9 (79.7-84.1) |
|  | Borderline | 5.8 (4.4-7.1) | 8.9 (7.2-10.6) |
|  | Abnormal | 6.8 (5.4-8.3) | 9.2 (7.6-10.9) |
|  | Total | 100.0 | 100.0 |
| Yes | Normal | 58.2 (45.0-71.5) | 55.4 (41.8-69.0) |
|  | Borderline | 19.2 (8.0-30.4) | 14.2 (4.7-23.7) |
|  | Abnormal | 22.5 (10.9-34.2) | 30.3 (17.8-42.9) |
|  | Total | 100.0 | 100.0 |
| 4-5 problem behaviours | **Emotional problems** |  |  |
| No | Normal | 75.7 (73.2-78.2) | 89.3 (87.6-91.0) |
|  | Borderline | 9.4 (7.8-10.9) | 5.7 (4.4-6.9) |
|  | Abnormal | 14.9 (13.0-16.9) | 5.0 (3.8-6.2) |
|  | Total | 100.0 | 100.0 |
| Yes | Normal | 46.8 (33.9-59.7) | 69.0 (56.1-82.0) |
|  | Borderline | 4.7 (0.1-9.4) | 4.9 (0.0-11.7) |
|  | Abnormal | 48.4 (35.8-61.1) | 26.0 (13.9-38.2) |
|  | Total | 100.0 | 100.0 |

CI – Confidence interval

| Table S3. Prevalence of 4-5 problem behaviours for selected socio-demographic characteristics for children 11-17 years | |
| --- | --- |
|  |  |
|  | Per cent (95% CI) |
| Age group - |  |
| 11-15 years | 3.3 (2.4-4.2) |
| 16-17 years | 5.5 (4.3-6.7) |
| Highest level of parent/carer education - |  |
| Bachelor degree or higher | 4.7 (3.4-6.0) |
| Diploma or Cert III/IV | 3.0 (2.1-4.0) |
| Year 11 or 12 | 3.4 (1.5-5.3) |
| Year 10 or below | 5.7 (2.7-8.6) |
| Parent/carer labour force status - |  |
| Both carers employed | 3.4 (2.5-4.3) |
| One carer employed, one carer not in employment | 4.8 (2.8-6.8) |
| Both carers not in employment | 8.6 (2.3-15.0) |
| Sole carer employed | 3.9 (2.3-5.5) |
| Sole carer not in employment | 3.5 (0.9-6.1) |
| Housing tenure - |  |
| Owned outright | 2.6 (1.0-4.1) |
| Owned with a mortgage | 3.5 (2.6-4.4) |
| Rented – public housing | 6.5 (2.4-10.6) |
| Rented – other | 5.3 (3.5-7.1) |
| Annual household income - |  |
| Less than $52,000 | 4.4 (2.8-6.0) |
| $52,000-$129,000 | 3.6 (2.6-4.6) |
| $130,00 or more | 4.3 (2.9-5.8) |
| Child’s country of birth - |  |
| Australia | 3.7 (3.0-4.5) |
| Overseas | 5.0 (2.6-7.4) |
| Family type - |  |
| Original family | 3.8 (2.9-4.7) |
| Step family | 3.7 (0.7-6.7) |
| Blended family^1^ | 4.4 (1.6-7.1) |
| Long parent family | 3.9 (2.5-5.3) |
| Level of remoteness - |  |
| Major cities of Australia | 4.6 (3.6-5.5) |
| Inner regional Australia | 2.8 (1.6-4.0) |
| Outer regional Australia | 2.6 (0.2-5.0) |
| Remote Australia | 3.7 (0.0-8.8) |

CI – Confidence interval

^1^ Blended families include those with 2 or more children, at least one of whom is the natural or adopted child of both parents, and at least one who is the step child of one of them.

| Table S4. Prevalence of risk factors by level of problem behaviours for children and adolescents^1^ | | |
| --- | --- | --- |
|  |  |  |
| Risk factors | **With < 4 problems** | **With 4-5 problems** |
|  | Per cent (95%CI) | Per cent (95% CI) |
| K10 - very high level | 3.3 (2.4-4.3) | 19.4 (7.7-31.1) |
| Major depressive disorder^2^ | 6.9 (6.0-7.8) | 25.4 (17.3-33.5) |
| Suicide attempt^3^ | 1.6 (1.2-2.0) | 14.1 (7.2-21.1) |
| Current alcohol user | 17.5 (15.7-19.2) | 29.9 (21.4-38.4) |
| Binged on alcohol | 12.1 (10.7-13.4) | 22.2 (14.8-29.5) |
| Ever used cannabis | 11.0 (9.5-12.4) | 24.8 (17.0-32.7) |
| Other drug user^4^ | 4.2 (3.4-5.1) | 10.3 (4.7-15.8) |
| Ever smoked tobacco | 9.5 (8.2-10.9) | 17.1 (9.9-24.3) |
| Current smoker | 6.9 (5.7-8.1) | 13.1 (7.0-19.2) |
| Ever had sex | 14.5 (12.9-16.1) | 23.2 (15.8-30.6) |
| Used services^5^ | 17.0 (15.3-18.8) | 36.4 (26.9-46.0) |

^1^ Age ranges for risk factors: 11-17 yrs – K10, major depressive disorder, self-esteem, family functioning; 12-17 yrs – self-harm, suicide attempt; 13-17 yrs – current alcohol user, binged on alcohol, current smoker, ever used cannabis, other drug user, ever had sex, used services.

^2^ Youth-reported

^3^ In previous 12 months

^4^ Includes using prescription drugs for non-medical purposes; ecstasy; amphetamines and methamphetamines; cocaine; hallucinogens such as LSD; inhalants such as petrol, glue, aerosols, paint, solvents or nitrous; heroin; steroids; GHB or ketamine

^5^ Youth-reported use of any service for emotional or behavioural problems in the past 12 months
